# Monitoring of Epidermal Growth Factor Receptor Tyrosine Kinase Inhibitor-Sensitizing and Resistance Mutations in the Plasma DNA of Patients With Advanced Non–Small Cell Lung Cancer During Treatment With Erlotinib

**DOI:** 10.1002/cncr.28964

**Published:** 2014-08-07

**Authors:** Boe S Sorensen, Lin Wu, Wen Wei, Julie Tsai, Britta Weber, Ebba Nexo, Peter Meldgaard

**Affiliations:** 1 Department of Clinical Biochemistry, Aarhus University HospitalAarhus, Denmark; 2 Department of Genomics and Oncology, Roche Molecular Systems IncPleasanton, California; 3 Department of Oncology, Aarhus University HospitalAarhus, Denmark

**Keywords:** epidermal growth factor receptor (EGFR) mutations, plasma DNA, erlotinib, lung cancer, resistance

## Abstract

**Background:**

The feasibility of monitoring epidermal growth factor receptor (EGFR) mutations in plasma DNA from patients with advanced non–small cell lung cancer (NSCLC) during treatment with erlotinib and its relation to disease progression was investigated.

**Methods:**

The amount of EGFR-mutant DNA was tested in plasma DNA from patients with advanced NSCLC with allele-specific polymerase chain reaction assays. Blood samples from 23 patients with adenocarcinoma of NSCLC that carried tyrosine kinase inhibitor-sensitizing EGFR mutations were taken immediately before treatment with erlotinib. Additional blood samples were taken at timed intervals until erlotinib treatment was withdrawn.

**Results:**

The amount of plasma DNA with sensitizing EGFR mutations was found to be reduced after the first cycle of erlotinib treatment in 22 of 23 patients (96%). No patients presented with the resistant T790M mutation in the pretreatment sample, but at the time of disease progression the mutation was detected in plasma from 9 patients (39%). The quantitative data from the current study demonstrated that when a T790M mutation emerged in the blood it was accompanied by an increase in the original sensitizing EGFR mutation. When T790M was detected, it was found to be present in all subsequent blood samples from that patient. Most interestingly, the results of the current study demonstrated that monitoring the EGFR mutations in the blood allows for the detection of the T790M mutation up to 344 days before disease progression is clinically evident (range, 15-344 days).

**Conclusions:**

The results of the current study demonstrated that serial monitoring of EGFR mutations in plasma DNA is feasible and may allow for the early detection of resistance mutations. These results warrant further studies to explore the clinical usefulness of such analysis.

## Introduction

Tyrosine kinase inhibitors (TKI) targeting the epidermal growth factor receptor (EGFR) represent a promising new group of anticancer agents for the treatment of patients with non–small cell lung cancer (NSCLC). These include erlotinib and gefitinib and it has been demonstrated that a group of mutations centered at the ATP-binding pocket of EGFR confer sensitivity to these agents by enhancing the binding of the TKI at the expense of ATP.^1^–^3^ The majority of these sensitizing mutations are a group of deletions in exon 19 and a point mutation in exon 21, the L858R mutation. However, for nearly all patients who initially respond, resistance develops and the disease progresses. This is often associated with the appearance of the T790M resistance mutation in EGFR.^4^,^5^ This mutation causes resistance by increasing the binding affinity of ATP compared with the TKI.^6^

It has been demonstrated that plasma DNA from patients with cancer contains DNA originating from the tumor.^7^–^10^ Comparison of sensitizing EGFR mutations found in biopsies and plasma DNA from the same patients has been performed and demonstrates varying degrees of correlation.^11^–^14^ The plasma DNA offers an opportunity to monitor the presence of EGFR mutations during the treatment of patients with lung cancer and to identify the emergence of resistance mutations.

Recently, it has been demonstrated in a few patients with different types of cancer (including a single patient with lung cancer) that the amount of sensitizing mutation as well as the development of the T790M resistance mutation can be identified by sequencing the plasma DNA.^15^ Detection of resistance mutations in plasma may prove to be of major importance in the clinical setting, because patients may benefit from adjustments in the treatment regimen.

In the current study, we monitored EGFR mutations during treatment with erlotinib in a group of patients with NSCLC, all of whom demonstrated sensitizing EGFR mutations in plasma DNA before the initiation of treatment with erlotinib. We demonstrated that the T790M resistance mutation was absent from the pretreatment sample but appeared in some but not all patients.

## Materials and Methods

### Patients and Blood Sample Collection

An unselected cohort of 199 patients with adenocarcinoma was included in the current study from October 2008 to December 2012 (95% with adenocarcinoma and 5% with adenosquamous carcinoma). The patients were all white except for one who was of Asian origin. There was an equal distribution of males and females (51% and 49%, respectively) and 9% of the patients were never-smokers. The overall concordance of EGFR mutation status in plasma and tumor biopsy specimens was 91%.^14^ A total of 23 patients had a sensitizing mutation in the pretreatment blood sample and represent the current study cohort. Of the 23 patients, 9 were men and 14 were women, all of whom were white, with a mean age of 65 years (range, 46-85 years) and a performance status (WHO) of 0 in 6 patients, 1 in 9 patients, and 2 in 8 patients. All patients had adenocarcinomas, with the tumor staged as II and III in 2 patients and IV in 21 patients (TNM 7th edition). All patients were treated with first-line treatment with standard chemotherapy (carboplatin [area under the curve, 5] intravenously and oral vinorelbine at a dose of 60-80 mg/kg) and erlotinib as second-line treatment (at a dose of 150 mg daily). Treatment was terminated as a consequence of disease progression, patient death, or unacceptable side effects. Patients underwent computed tomography scans every 3 months until disease progression. Blood samples were collected at every visit, which was approximately every 4 weeks for the first 3 months and every 6 weeks thereafter. Blood samples were taken during erlotinib treatment until disease progression, which was defined according to the Response Evaluation Criteria in Solid Tumors (RECIST; version 1.0) guidelines for measuring solid tumors^16^ combined with the medical judgment of the treatment benefit. Time to disease progression was calculated as the time from the initiation of erlotinib treatment until disease progression. Response data were available from 20 of the 23 patients. Of these, a response rate of 55 % was observed and a disease control rate of 90% was noted. The median time to disease progression was 6.7 months.

Informed consent was obtained from all patients. The project was approved by the Central Denmark Region Committees on Biomedical Research Ethics (M-20080012) and reported to http://ClinicalTrials.gov (NCT00815971).

### Blood Sample Collection and Detection of EGFR Mutations

Blood samples were serially collected in collection tubes containing ethylenediamine tetraacetic acid before treatment with erlotinib (within 2 days). Centrifugation was performed at 1000 revolutions per minute for 15 minutes and plasma was removed and stored at −80°C. Two mL of the plasma was used for each test. DNA was isolated with the cobas DNA Sample Preparation kit (Roche Molecular Systems, Pleasanton, Calif). For use with this kit, proteinase K, wash buffer I, and wash buffer II were prepared following the manufacturer's instructions. The plasma was mixed with 250 µL of proteinase K and 2 mL of DNA polybrominated biphenyls (binding buffer) and incubated at room temperature for 30 minutes. Then, 500 µL of isopropanol was mixed with the lysate and the mixture transferred into the High Pure Extender Assembly (Roche Molecular Systems). The High Pure Extender Assemblies were centrifuged at 4000 ×g for 1 minute. The extenders were removed from the filter, and the filters were placed in new collection tubes and washed with wash buffer I and wash buffer II according to the manufacturer's instructions. The DNA was eluted in 100 µL of DNA elution buffer.

The cobas EGFR Blood Test (currently in development by Roche Molecular Systems) was used for mutation detection. The test is designed to detect G719A/C/S in exon 18; 29 deletions in exon 19; S768I, T790M, and 5 insertions in exon 20; and L858R and L861Q in exon 21. A total of 42 EGFR mutations can be detected. For each polymerase chain reaction, 25 µL of the DNA eluate was used. Polymerase chain reactions were run on the cobas z 480 analyzer with cobas 4800 SR2 System Software (version 2.0) and EGFR Blood Analysis Package Software (in development) (Roche Molecular Systems). Amounts of mutant EGFR DNA were estimated by comparison of crossing point values with a serial of dilutions of genomic DNA mixed with known copies of EGFR mutants.

## Results

A pretreatment blood sample was taken 0 to 2 days before the initiation of treatment with erlotinib. A total of 23 patients both had a sensitizing EGFR mutation in the pretreatment blood sample and continued treatment long enough to have additional blood samples collected during erlotinib treatment. From these patients, a blood sample had been taken at the time of every visit to the clinic for routine examination (every 4 weeks for the first 3 months and every 6 weeks thereafter). Plasma DNA from all blood samples collected during treatment was investigated for the amount of EGFR-mutated DNA. Quantitative data for the amount of mutated DNA present in plasma could be retrieved from all patients except one who carried a L861Q EGFR mutation. This patient demonstrated mutations only in the pretreatment sample.

**Figure 1:**
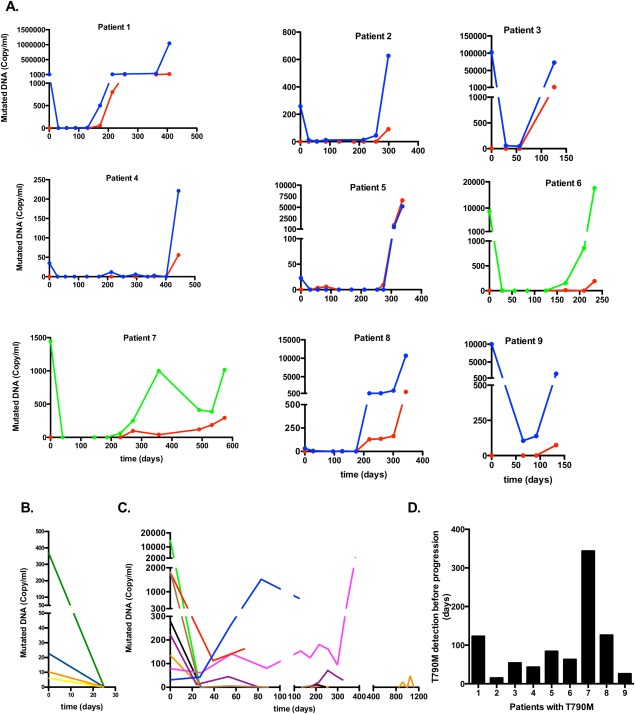
Amount of mutated epidermal growth factor receptor (EGFR) DNA is shown in the plasma isolated from patients with lung cancer who were treated with erlotinib. (A) Results from patients harboring the EGFR resistance mutation T790M (9 patients) are shown. Red line indicates T790M mutation; blue line, EGFR-sensitizing deletion in exon 19; green line, EGFR-sensitizing mutation L858R. (B) Patients in whom EGFR mutations were recorded only in the pretreatment sample (5 patients) are shown. Yellow line indicates 2 patients with identical values. (C) Patients with EGFR-sensitizing mutations present in >1 sample (8 patients) are shown. In panels A to C, the x-axis is the time from the initiation of treatment in days and the y-axis is the estimated copy number for the indicated mutations. Quantitative amounts of tumor DNA are shown for 22 of the 23 patients. The single patient with an L861Q mutation could not be quantitated. For clarity, the presentation of the different patients was done with different scaling of the x-axis and the y-axis. (D) The time point for the earliest identification of the T790M mutation in the blood and the time point for the identification of disease progression with scanning (Response Evaluation Criteria In Solid Tumors [RECIST] criteria) were determined. The figure demonstrates how many days earlier the T790M mutation could be detected in the blood before disease progression could be identified by scanning.

The results demonstrated that the levels of plasma DNA containing sensitizing EGFR mutations decreased in all patients except one after the first 4 weeks of erlotinib treatment (Table 1). For 13 of the patients (57%), the amount of mutated DNA was reduced to an undetectable level by erlotinib treatment and 6 of these patients remained without detectable mutations for the duration of the study (Table 1). Seventeen of the patients demonstrated sensitizing mutations in at least 1 posttreatment sample, and the values fluctuated during the course of treatment, as depicted in Figure 1 (panels A-C). The type of sensitizing mutation remained the same for each patient throughout the study period.

**Table 1:** Summary of Mutations in Plasma DNA

Patient	Pretreatment Mutation	Reduction or Increase in Mutated DNA After First Erlotinib Treatment^a^	Mutation at Time of Disease Progression	Time to Disease Progression, Days
1	Ex19 Del	R*	Ex19Del + T790M	276
2	Ex19Del	R	Ex19Del + T790M	341
3	Ex19Del	R	Ex19Del + T790M	140
4	Ex19 Del	R*	Ex19Del + T790M	369
5	Ex19Del+G719X	R*	Ex19Del + G719X + T790M	343
6	L858R	R	L858R + T790M	232
7	L858R	R*	L858R + T790M	443
8	Ex19Del	R	Ex19Del + T790M	369
9	Ex19Del	R	Ex19Del + T790M	161
10	Ex19Del	I	Ex19Del	133
11	Ex19Del	R*	Ex19Del	208
12	L858R	R	L858R	110
13	Ex19Del	R	Ex19Del	350
14	Ex19Del	R	Ex19Del	28
15	L858R	R	L858R	383
16	Ex19Del	R*	None	170
17	Ex19Del	R*	None	69
18	Ex19Del	R*	None	373
19	Ex19Del	R*	None	12
20	L861Q	R*	None	84
21	Ex19Del	R*	None	63
22	L858R	R*	None	205
23	Ex19 Del	R*	None	1007

Abbreviations: I, increase; R, reduction.

a R* represents the absence of a mutation after the first round of treatment.

In 9 of the patients, the resistance mutation T790M appeared in the blood during erlotinib treatment (Fig. 1A). The T790M mutation was always present together with the original sensitizing EGFR mutation and the appearance of T790M always occurred together with an increase in the amount of the sensitizing EGFR mutation (Fig. 1A). The quantitative measurements demonstrated that the T790M mutation continued to increase in all 9 patients until disease progression (Fig. 1A). This is in contrast to the patients who did not develop T790M mutations, in whom there was no trend toward an increase in the original sensitizing EGFR mutation noted (Figs. 1B and 1C). Once the T790M mutation was identified, it was found in all subsequent blood samples that patient. For a group of 5 patients, the sensitizing EGFR mutation was not found in any samples after the first exposure to erlotinib (Fig. 1B). Another group of 8 patients did not develop the T790M mutation and the originally detected sensitizing mutation was found to be present in ≥1 samples during erlotinib exposure (Fig. 1C).

Examination of the last blood sample taken before disease progression demonstrated that 9 patients had a T790M mutation combined with the sensitizing EGFR mutation, 6 patients had the sensitizing mutation in the absence of the T790M mutation, and 8 patients had neither the sensitizing nor T790M mutations.

Time to disease progression is shown in Table 1. The median time to disease progression was 341 days (range, 140-443 days) for the patients with the EGFR resistance mutation T790M (9 patients) compared with 152 days (range, 12-1005 days) for the remaining patients (14 patients). It is interesting to note that the T790M resistance mutation was identified in the blood between 15 and 344 days before disease progression was evident according to RECIST criteria (Fig. 1D).

## Discussion

Among patients with lung cancer, it has been shown that the effect of treatment with the EGFR inhibitor erlotinib is highest if the tumor cells have specific mutations in EGFR.^1^,^2^,^17^ In the current study, we examined EGFR mutations in plasma samples collected during erlotinib treatment from 23 patients with lung cancer in whom EGFR mutations were identified in the blood sample taken before the initiation of treatment. Plasma DNA was isolated from the blood samples and analyzed for 42 different EGFR mutations. Examination of the serially collected blood samples revealed several interesting findings. One of these was that the amount of the mutated tumor DNA in the first blood sample taken after erlotinib treatment decreased for 22 of the 23 patients. This suggests that erlotinib already has an effect at this early stage of treatment in the majority of these patients.

All 23 patients with lung cancer with mutated EGFR DNA in the pretreatment blood sample ultimately developed disease progression during treatment with erlotinib. Investigation of the blood samples taken during erlotinib treatment revealed considerable variation but also similarities among the patients, and 3 major groups could be identified. Nine patients had a T790M mutation combined with the sensitizing EGFR mutation, 6 patients had the sensitizing mutation in the absence of the T790M mutation, and 8 patients had neither the sensitizing nor T790M mutations.

The results of the current study demonstrated that the T790M mutation was not present in the blood before treatment with erlotinib in any of the 23 patients with sensitizing EGFR mutations noted in the pretreatment blood sample. This is in contrast to studies performed by another group, in which the T790M mutation was found in 35% of tumors with sensitizing EGFR mutations that were EGFR inhibitor-naïve.^18^ To the best of our knowledge, the reason for this apparent discrepancy is currently unclear, but might be caused by differences in sample material and/or the sensitivity of the assays used.

The ability to generate quantitative data for the EGFR-mutated plasma DNA allowed us to demonstrate that the appearance of the T790M mutation during treatment always was associated with an increase in the amount of the original sensitizing mutation. Furthermore, the T790M mutation continued to increase in all 9 patients until progression of the disease. This is in contrast to those patients who did not develop the T790M mutation, in whom there was no trend toward an increase in the original sensitizing EGFR mutation noted. Further research should be performed to identify the relevant mutations in these patients and follow them using blood samples. Once the T790M mutation was detected, it was identified in all subsequent blood samples from that patient.

It has been demonstrated that the T790M mutation can appear as a secondary mutation in tumor cells already harboring a sensitizing EGFR mutation.^5^ In the blood samples from those patients with the T790M mutation, the amount of T790M and EGFR-sensitizing mutations increased in parallel for some but not all patients. A likely explanation is that apart from the tumor cells with both sensitizing and T790M EGFR mutations, some of the patients had residual amounts of tumor cells that only possessed the sensitizing mutation.

Patients with T790M-mutated tumors have been characterized as a subgroup whose prognosis is relatively favorable with an indolent type of disease progression.^19^ This is in agreement with the results from the current study among a limited number of patients, in whom we demonstrated that the median time to disease progression more than doubled compared with the patients without T790M mutations. Most interestingly, when examining the appearance of the T790M mutation in the serial blood samples, we demonstrated that the mutation can be detected in the plasma DNA up to 344 days before disease progression is clinically evident by RECIST criteria (range, 15-344 days before). The ability to detect T790M might be important for the identification of those patients who are eligible for treatment with emerging new third-generation inhibitors that target T790M-mutated EGFR.

The results of the current study demonstrated that examination of a plasma sample allows for the quantitation of EGFR mutations and that EGFR resistance mutations may be detected well in advance of disease progression. These findings may have implications for the identification of patients who might benefit from an altered treatment strategy when T790M resistance is already identified in the blood. Future studies in a larger patient cohort will indicate whether these patients might benefit from TKIs or other agents that also inhibit the T790M-mutated EGFR.
